# Usability of Speculum-Compatible Injection Devices for Administering Ethyl Cellulose-Ethanol Ablation to Treat Cervical Neoplasia in Low- and Middle-Income Countries

**DOI:** 10.1007/s10439-025-03799-8

**Published:** 2025-07-21

**Authors:** Taya Lee, Vené Richardson-Powell, Jason Chen, David Garvey, Venkata Sarojasamhita, Kevin Aroom, Martha O. Wang, Brian Crouch, Nimmi Ramanujam, Julie Hurvitz, Jenna Mueller

**Affiliations:** 1 Department of Bioengineering, University of Maryland, College Park, MD USA; 2 Robert E. Fishell Institute for Biomedical Devices, University of Maryland, College Park, MD USA; 3 Department of Biomedical Engineering, Duke University, Durham, NC USA; 4 Calla Health Foundation, Durham, NC USA; 5 Duke Global Health Institute, Duke University, Durham, NC USA; 6 Department of Pharmacology and Cancer Biology, Duke University, Durham, NC USA; 7 Department of Obstetrics, Gynecology & Reproductive Science, University of Maryland School of Medicine, Baltimore, MD USA; 8 Marlene and Stewart Greenebaum Cancer Center, University of Maryland School of Medicine, Baltimore, MD USA

**Keywords:** Injection device, Cervix, Cervical neoplasia, Device usability, Ethanol ablation

## Abstract

**Purpose:**

Current treatments for cervical neoplasia are often inaccessible in low- and middle-income countries (LMICs), which contributes to high cervical cancer mortality. We previously developed a low-cost ablative therapy using ethanol mixed with ethyl cellulose (EC) to a form an ethanol-retaining gel that reduces injection leakage. To optimize delivery of EC-ethanol into the cervix, we developed and compared three speculum-compatible injectors that each address clinical challenges: 1) a single needle injector, which contained an adjustable depth stop to control the depth of injection, 2) a multi needle injector, which injected three locations in the cervix simultaneously, and 3) an extender injector, which included a needle extender.

**Methods:**

The variability in EC-ethanol injections was evaluated through bench top and *ex vivo* swine testing. Usability testing was performed by gynecology (GYN) providers who used each device in a custom pelvic model.

**Results:**

Both the extender and single needle devices led to consistent ejection volumes in benchtop tests with no variability between injections. All devices achieved spherical depots with minimal leakage in *ex vivo* tests. In usability testing, 65% of GYN providers preferred the extender device, which achieved significantly shorter injection times in the custom pelvic model compared to other injectors.

**Conclusion:**

While all devices met clinical constraints, the extender device was preferred by clinicians and achieved repeatable injection distributions. This work presents a clinically informed low-cost intracervical delivery method for LMICs. Future work will include validating performance in clinical trials and assessing feasibility in clinical settings to advance global cervical neoplasia treatment.

**Supplementary Information:**

The online version contains supplementary material available at 10.1007/s10439-025-03799-8.

## Introduction

In 2020, over half a million cases of cervical cancer were diagnosed worldwide and a quarter of a million women died from the disease [1]. Of these deaths, 85% occurred in low- and middle-income countries (LMICs) [2]. Virtually all cervical cancers are caused by human papillomavirus (HPV) infection, which can lead to the growth of abnormal, precancerous cells on the surface of the cervix known as cervical neoplasia (CIN), the precursor to cervical cancer [3, 4]. CIN is subclassified based on the depth of invasion and can reach up to 5 mm deep and up to 2 cm wide [5]. Detection and treatment of CIN provides an opportunity to prevent progression to invasive cervical cancer, thus improving outcomes [4].

In high-income countries, CIN is commonly treated with a surgical procedure called a Loop Electrosurgical Excision Procedure (LEEP). LEEP requires a reliable source of electricity, costs over $3,700 for machine and procedure materials, and requires an OB-GYN to perform [6, 7]. Because of these challenges, the World Health Organization recommends ablative therapies, such as cryotherapy and thermocoagulation, for CIN treatment in LMICs, which can be performed by primary care providers [8]. Cryotherapy uses high-quality gas to freeze lesions which subsequently leads to necrosis. However, this method is still moderately expensive (over $1700 for machine and equipment), requires large 50–70 pound gas tanks that can be difficult to procure and transport in LMICs, and only reaches depths up to 3.5 mm in tissue, preventing it from successfully treating deeper lesions [9]. Another ablation technique on the market is thermocoagulation which uses heat to ablate the area of interest. This method is also moderately expensive ($2500–$5,370 for machine and probes), requires a reliable electricity source, and similarly treats superficial lesions (~3.5 mm) [6]. Thus, there exists an opportunity to develop inexpensive and easy-to-use therapies for patients with deeper lesions in LMICs.

To address this unmet need, our group developed a low-cost, accessible therapy that combines a biocompatible polymer, ethyl cellulose (EC), with ethanol to induce necrosis when injected into tissue [10]. Upon injection to aqueous environments, EC-ethanol crosslinks, forming a gel that sequesters ethanol at the injection site, controlling the zone of necrosis. The therapeutic efficacy of EC-ethanol was first evaluated in a hamster cheek pouch model of oral cancer; results indicated that 3% EC-ethanol (EC:ethanol, w:v) delivered at a slow injection rate of 10 mL/hr significantly stunted tumor growth when compared to pure ethanol injections [10]. Next, a larger range of EC concentrations and infusion rates were evaluated in *ex vivo* swine liver. Fluid leakage remained low for 6% EC-ethanol compared to 3% EC-ethanol and pure ethanol, regardless of infusion rate, indicating that if EC concentration was ≥ 6%, injection rate was less critical to control [11]. When EC-ethanol was injected into excised swine cervices, orienting the needle bevel radially toward the outer edge of the cervix decreased fluid leakage into the endocervical canal [12]. Additionally, inserting the needle to a depth of ≥ 13 mm minimized backflow to the tissue surface, and injection volumes ≤ 3 mL of 6% EC-ethanol minimized fluid leakage away from the injection site [13]. Taken together, it is important to control the EC concentration, injection depth, and injection volume to reduce leakage during EC-ethanol administration.

To control key injection parameters during EC-ethanol injection into the cervix, we developed three speculum-compatible injector prototypes, a single needle injector, a multi needle injector, and an extender injector. These three devices were created to follow a human-centered design approach by addressing clinical constraints, providing different designs, and having clinicians give feedback on different aspects (Table 1). The single needle injector is minimalistic in its design–it contains a needle, a needle extender that has been over-molded with a custom grip, and an adjustable needle depth stop to control the insertion depth [13]. Conversely, the multi needle injector is bulkier and more complex; it can perform three injections simultaneously to ablate lesions that cover the entire cervical face, and the injections are driven by an air pressure canister. Lastly, the extender injector is made from commercially available components; it includes a needle, needle extender, and an adjustable needle stopper, which was originally designed for controlling the depth of dermatological injections. Here, the performance and usability of these three injectors were compared through bench testing, testing in *ex vivo* swine cervices, and usability assessments by gynecology (GYN) providers. Determining which speculum-compatible injector was preferable represents a critical step toward translating EC-ethanol ablation to women with CIN in LMICs.

**Table 1:**
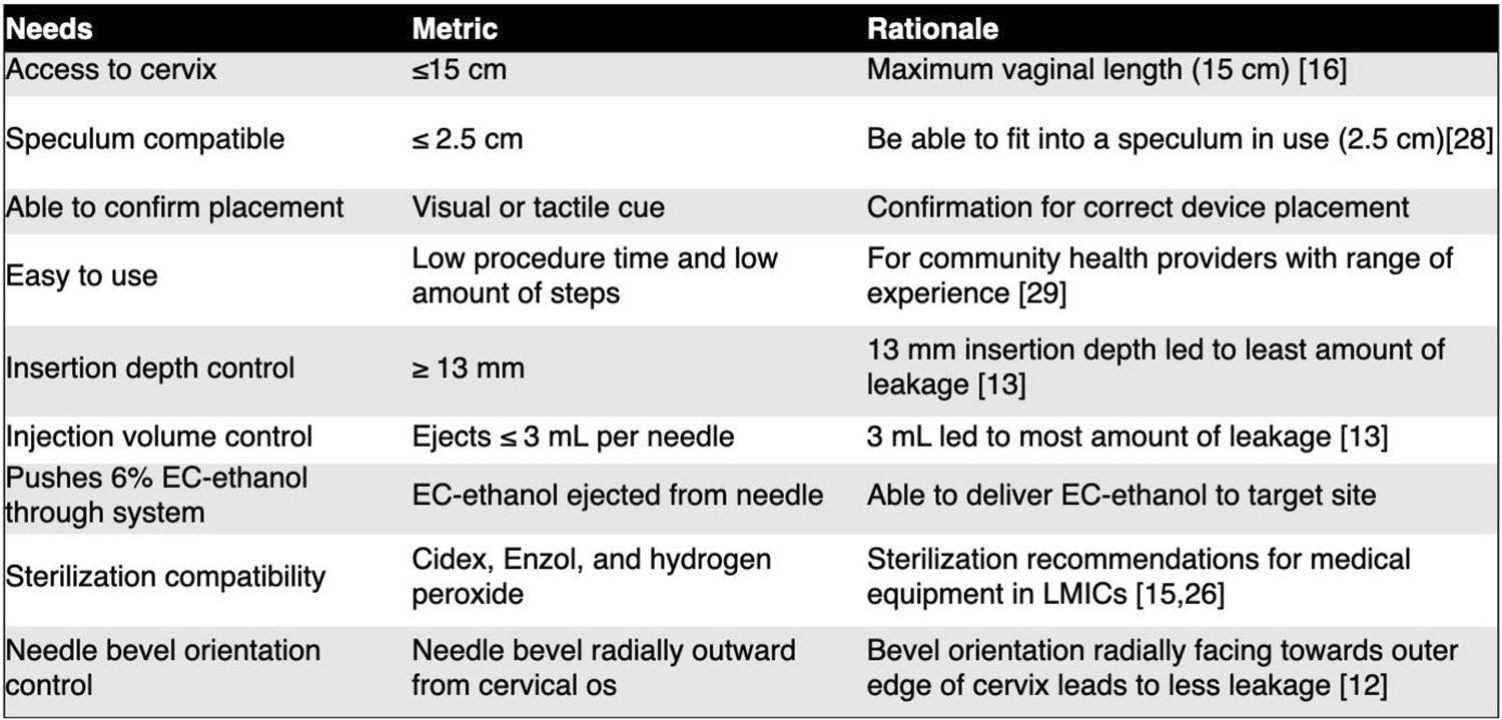
Device constraints

## Materials and Methods

### Injector Designs

**Fig. 1:**
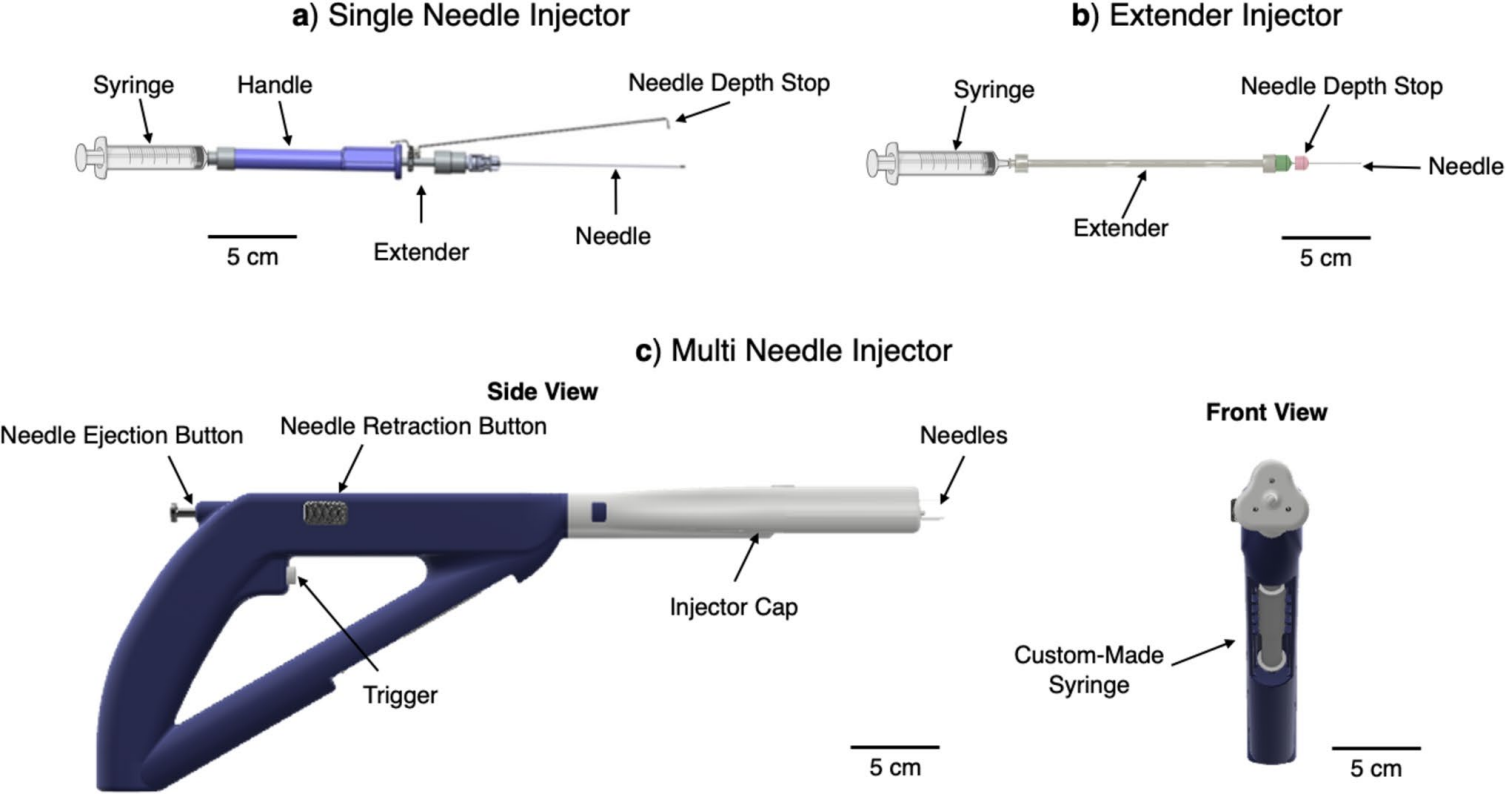
Renderings of three devices: (**a**) single needle injector, (**b**) extender injector, (**c**) multi needle injector (showing both side and front views). Scale bars = 5 cm. Syringe created with BioRender.com

The single needle injector was previously described in Adhikari et al. [13]. Briefly, the single needle injector design (Fig. 1a and Table 2) includes a 22G 8.9 cm-long needle (4871835, Becton, Dickinson and Company, Franklin Lakes, NJ) connected to a needle extender that is over-molded with a custom grip to enable ease of use by clinicians for single locations in the cervix at a time. The single needle device was designed around the 8.9 cm 22G needle currently used to inject indocyanine green (ICG) into the cervix for lymph node mapping [14]. Since needle depth is important to control and minimize backflow, a needle depth stop was incorporated into the device, which can be adjusted by sliding the mechanism forward or backward and then locking in place at the desired depth through tightening a set screw [13]. This device was connected to a standard 3-mL syringe (309657, Becton, Dickinson and Company, Franklin Lakes, NJ). The single needle also incorporates both reusable and single-use components. The needle and syringe are single-use and are disposed after each procedure, while all remaining parts can be sterilized using submersion sterilization, which is commonly used in resource-limited healthcare settings [15].

**Table 2:**
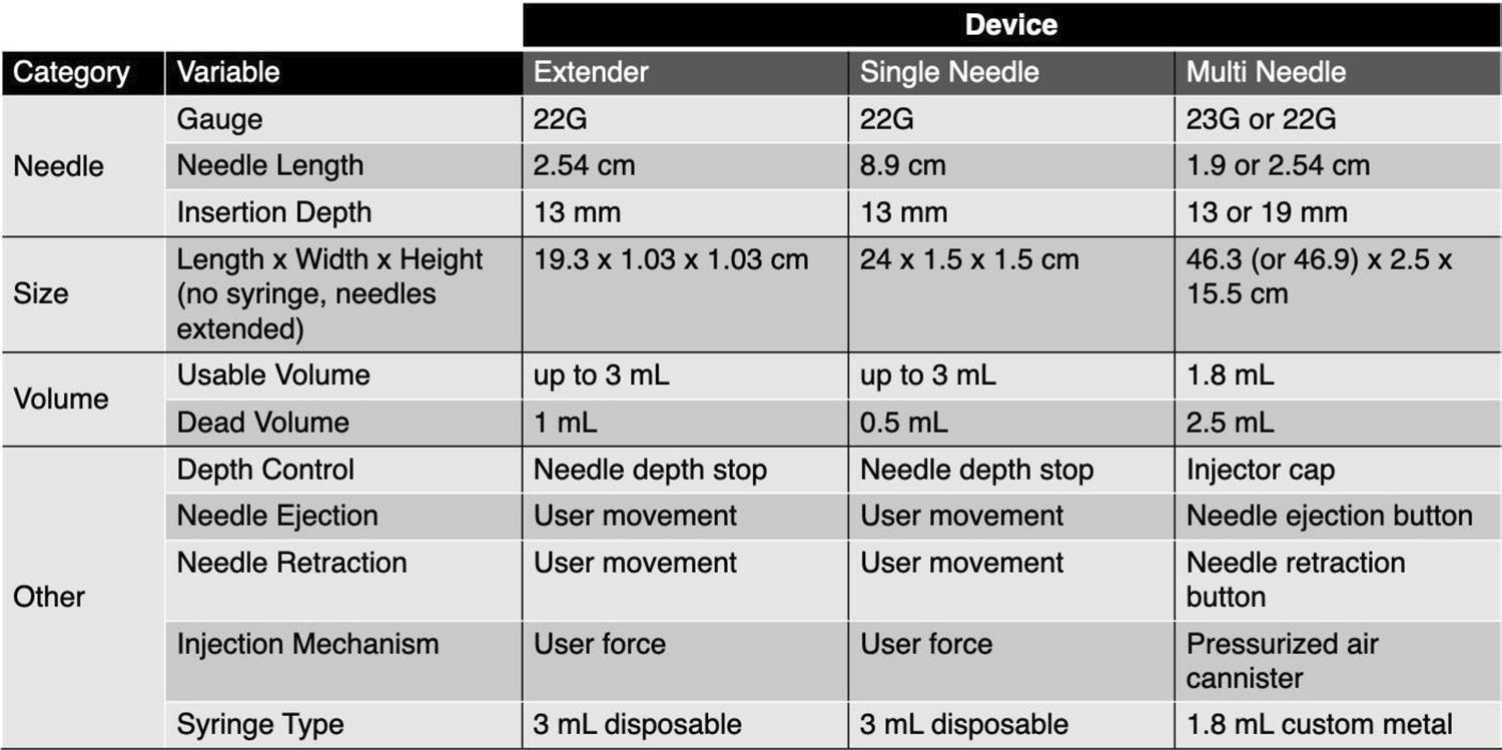
Specifications of the three speculum-compatible devices

We also developed a multi needle injector that can perform three injections simultaneously to fully ablate lesions that cover the entire cervical face quickly (like cryotherapy and thermocoagulation). Prior studies have shown that large individual injection volumes increase fluid leakage from injection sites [13]. Thus, multiple injections (i.e., 3) are required to cover the entire cervical face. We hypothesized that a device that simultaneously injects at three points would reduce procedure time while decreasing injection site leakage. The multi needle injector device is controlled by air pressure to simultaneously push EC-ethanol through three equidistant needles (Fig. 1c and Table 2). Either 2.54 cm-long 22G bevel needles or 1.9 cm, 23G bevel needles were used with this device (26407, Exelint International, Company, Redondo Beach, CA). The device controls the injection volume (0.6 mL per needle for a total of 1.8 mL), injection rate, and injection depth. Specifically, injection rate is controlled by an air canister (AVCO2TANK-20OZ, Action Village, RoadSimi Valley, CA) that pressurizes the device’s tubing system. This pressure pushes the plunger of a customized, reusable metal syringe that ejects EC-ethanol from the device. To pressurize the air canister, the air canister can be attached to a bike pump (VFP-004, Vibrelli, Bondi, New South Wales, Australia), which was selected because it is a low-cost, portable option that does not require electricity making it more amendable to LMIC environments. An input pressure of 90 psi was used for the multi needle injector because ejection rate per needle was similar to the single needle and extender devices (Supplementary Fig. 1). To control injection depth, the removable cap of the device was used to expose 13 to 19 mm of the needles (depending on needle length). The multi needle device has the least number of single-use parts (only its needles are single-use). The removable injector cap, which goes into the speculum, is submersion sterilization compatible. The handle of the device and custom-made syringe, which does not go into the speculum, can be wiped down with a disinfectant.

Lastly, we assembled a third injector made from commercially available components, which is referred to as the extender needle injector (Fig. 1b and Table 2). Like the single needle injector, the extender device is designed to inject at single locations in the cervix at a time. The extender needle injector includes a 2.54 cm-long 22G bevel needle (305155, Becton, Dickinson and Company, Franklin Lakes, NJ), a needle stopper to control the depth of the injection, a 15.2 cm-long stainless-steel needle extender (965196, Sklar, West Chester, PA), and a standard 3-mL syringe (309657, Becton, Dickinson and Company, Franklin Lakes, NJ). The length was chosen to ensure full reach to various women’s cervices, which can vary from 6 to 15 cm back [16]. The 15.2 cm extender accommodates the furthest-reaching cervix for optimal usability. The extender is mostly made of single-use parts and can be sterilized using submersion sterilization. The needle, needle depth stop, and syringe all are single-use and disposed of between patients.

### Bench Testing

First, we evaluated the injectors through bench testing using 22G 2.54 cm needles for the multi needle and extender devices and a 22G 8.9 cm needle for the single needle device. The multi needle injector’s 1.8-mL custom syringe was fully loaded with 6% EC-ethanol. The single needle and extender devices were both loaded with 0.6 mL of EC-ethanol. 0.6 mL was chosen as an estimate of the ejection volume of one needle during a multi needle ejection; thus, 0.6 mL was used to enable comparison across the three devices. All three devices were then ejected into graduated microcentrifuge tubes (05408138, Thermo Fisher Scientific Inc., Waltham, MA) to determine the ejection volume. The ejection rate was determined by measuring the time to reach the set volume.

### Tissue Preparation

To evaluate the repeatability of the distribution volume obtained with each device, we performed injections in *ex vivo* swine cervices using previously described methods [13]. Briefly, swine reproductive tracts were obtained from Animal Biotech Industries (Doylestown PA). The cervix of the tract was sectioned into 2.54 cm sections and placed in 10% PBS until experiments were performed. Each section was injected within 6 hours of receipt.

### EC-Ethanol Preparation and Contrast Agent Preparation

To visualize EC-ethanol solutions in excised tissue, 200 proof ethanol (Koptec, King of Prussia, PA, USA) and EC (Sigma Aldrich, St. Louis, MO, USA) were mixed to create 6% EC-ethanol solutions (EC:ethanol, w:v). For tissue experiments, iohexol (Omnipaque, GE Healthcare, Chicago, IL) was mixed with the 6% EC-ethanol at 40 mg/mL. Iohexol is a CT contrast agent that has been optimized and used for EC-ethanol visualization in previous tissue studies [12]. For the benchtop and clinician usability studies, aliquots of 6% EC-ethanol with no iohexol were used (as no iohexol will be included in the clinical formulation).

### *Ex Vivo* Injection Procedure

A 3-mL syringe was loaded first with 200 proof ethanol to prime each device. 6% EC-ethanol-iohexol was then loaded into either a 3-mL syringe (for the single and extender devices) or into the custom syringe (for the multi needle device), and the syringe was loaded into the device. Then, EC-ethanol-iohexol was pushed through the device until a steady drip of the solution was visually observed. Needle(s) were manually inserted into the tissue orienting the bevel tip facing away from the endocervical canal. When needles were in place, the injection was initiated, and the time to complete the procedure was recorded.

### Imaging and Image Analysis

Imaging and image analysis were performed as previously described [13]. Briefly, the tissue was scanned in a micro-CT machine (Bruker SkyScan 1276, Billerica, MA), and step-and-shoot projections were acquired with a field view of 9.31 x 158.75 cm for a full 360̊ rotation (70 kV, 200 µA, 50 ms exposure time, 0.5 mm aluminum filter). NRecon (Bruker) reconstruction software was used to create three-dimensional images (post-alignment correction, ring correct, and beam-hardening correction were applied to each sample). Image segmentation of the EC-ethanol-iohexol distributions was then performed in CT Vox software (Bruker) by applying Renyi entropy thresholding and adjusting the threshold values to set as lower to highlight only the present EC-ethanol within the tissue. Subsequent quantification of the distribution volume was performed in 3D slicer (Kitware, Clifton Park, NY). Specifically, both the total distribution volume and normalized distribution volume (i.e., total distribution/known injection volume) were calculated.

### Usability Assessment

**Fig. 2:**
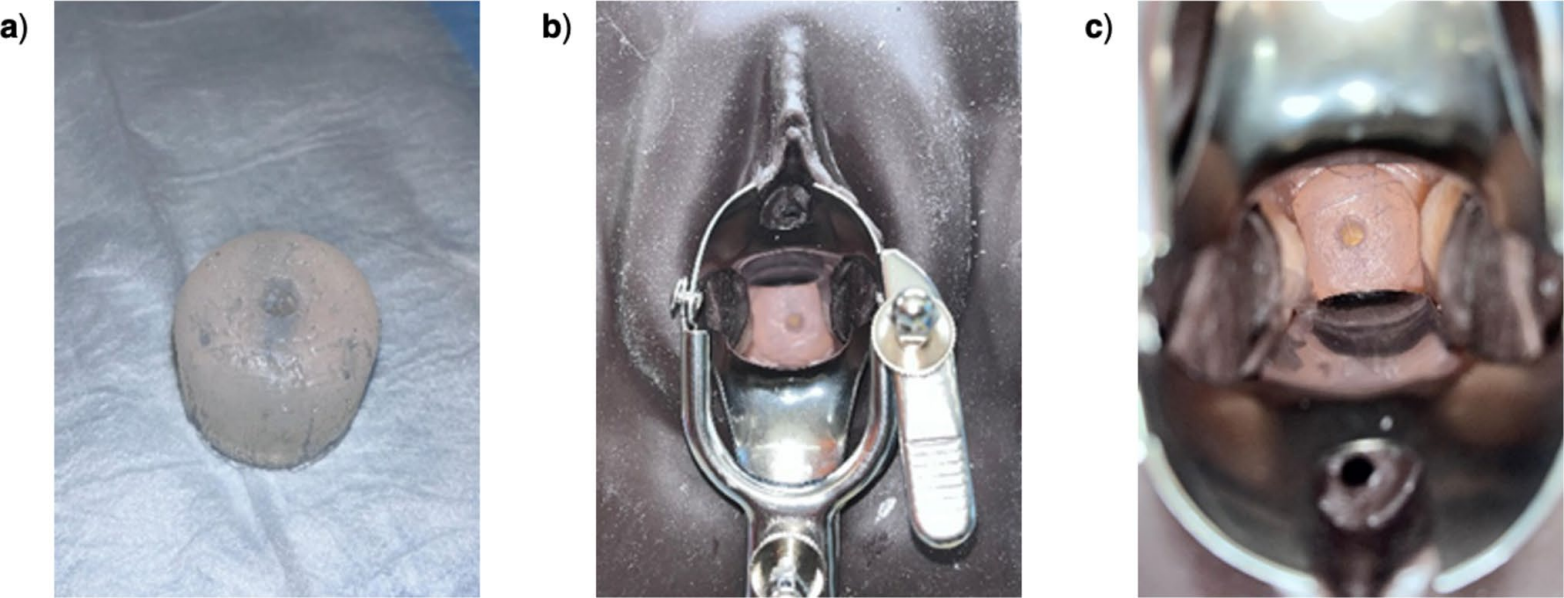
Customized pelvic model used by clinicians. **a** The custom silicone cervix, **b** pelvic model from physician’s view with silicone cervix inside, **c** a closer view of the custom silicone cervix inside of the pelvic model

The usability study was approved by the University of Maryland School of Medicine IRB panel (IRB: HP-00108639). To better understand user preference, GYN providers (residents and attendings) assessed each of the three injector devices in a custom pelvic model. The custom pelvic model included the Life/form Cervical Exam and Pap Smear Test Trainer from Nasco Healthcare (LF01231). The plastic cervix in the model was replaced with a human-sized cervix made from silicone rubber (Smooth On, Macungie, PA), which enabled providers to simulate injecting the cervix with EC-ethanol (Fig. 2). A demonstration of each device was given prior to the participant testing the device. Then, each clinician was asked to inject 6% EC-ethanol into the custom pelvic model using all three devices. The device order was randomized to prevent user bias. Each injection was timed by our research team. After completion of the procedures, clinicians completed a survey rating device usability and preference using Likert scales.

### Statistical Analysis

Sample size of *n* = 5–7 trials was performed for each parameter for all experiments unless otherwise indicated. In clinician usability studies, 13 clinicians tested each device and completed the survey. All 13 clinicians tested each of the three devices and completed post-procedure questionnaires. However, for the multi needle device, the procedure time for one clinician was not recorded due to loss of data. As a result, the average time analysis (Fig. 7a) for the multi needle injector includes data from 12 clinicians (*n* = 12), while the other two devices have data from all 13 clinicians (*n* = 13). All post-procedure questionnaire analyses (Fig. 5, 6, 7b) have *n* = 13 for all devices. Kruskal–Wallis (non-parametric ANOVA) or Wilcoxon rank sum tests were performed in MATLAB (MathWorks, Natick, MA) to assess if there were significant differences between groups. Rejection of the null hypothesis was determined by significance levels of *p* < 0.05.

## Results

**Fig. 3:**
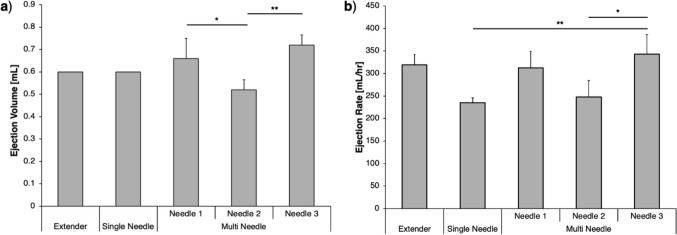
Benchtop validation experiments using 6% EC-ethanol. (**a**) Ejection volume and (**b**) ejection rate for the extender, single needle, and multi needle devices. Error bars indicate standard deviation (*p < 0.05 and **p < 0.005)

Benchtop experiments were done to validate each device and compare their performance before introducing tissue heterogeneity. The single needle and extender injector devices ejected 0.6 mL of EC-ethanol and showed zero variability in ejection volume between trials (Fig. 3a). Conversely, the multi needle’s three needles had significant variability in ejection volume when comparing the three needles to each other. Both needle 1 and 2 were significantly different than needle 3 (*p* = 0.043 and *p* = 0.001, respectively). During each experiment, the time to complete the ejection was also recorded. The extender’s ejection rate was modestly faster (319 mL/hr ± 23 mL/hr), but not significantly faster than the single needle injector (236 mL/hr ± 11 mL/hr) (Fig. 3b). The multi needle device had variability in ejection rate between each needle; the standard deviation of needle 1 and 2’s ejection rate was ± 36.5 mL/hr, whereas needle 3 was ± 42.8 mL/hr. Needle 3 was significantly faster than needle 2 (*p* = 0.017) and the single needle device (*p* = 0.008).

**Fig. 4:**
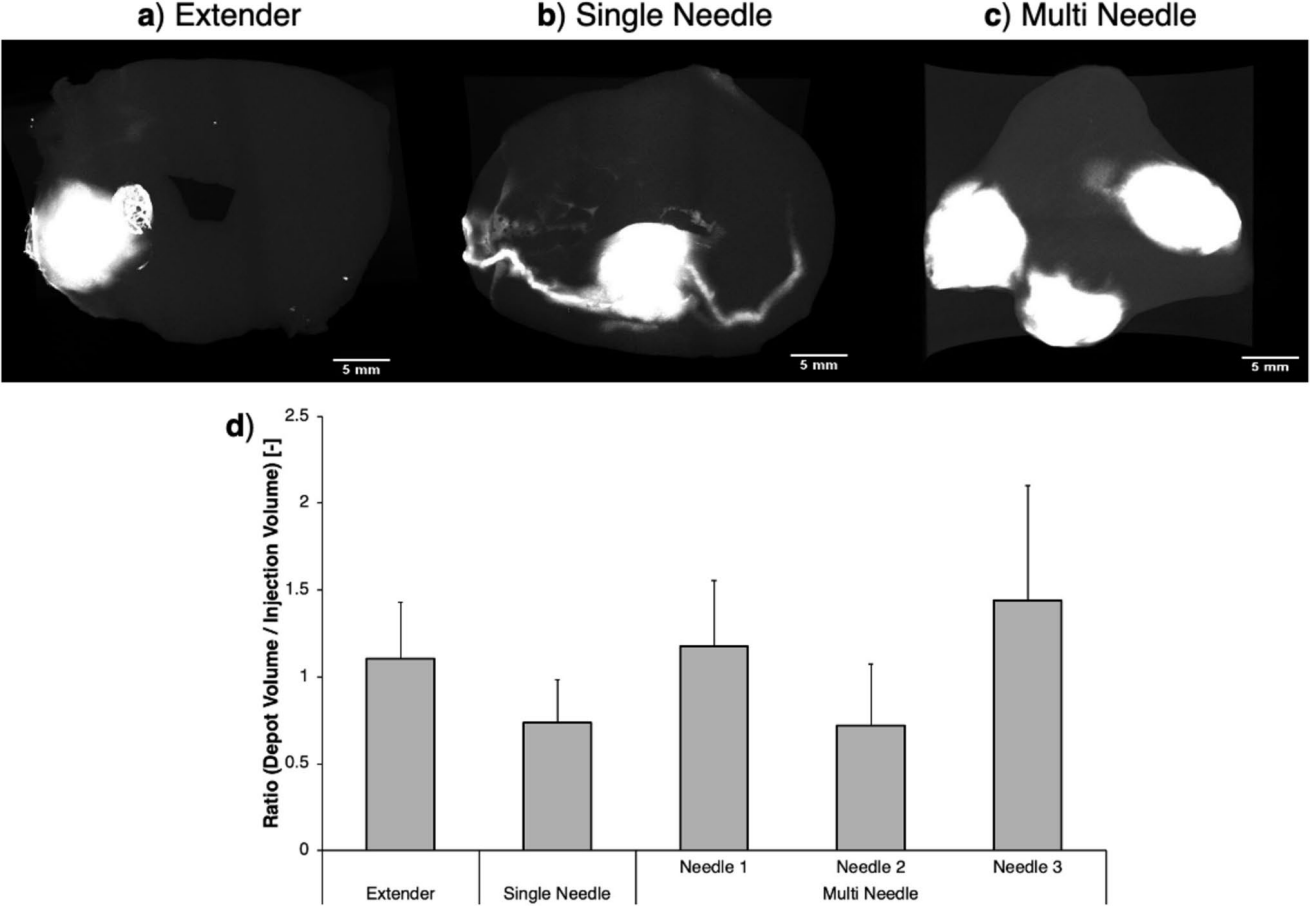
Reconstructed post-injection CT images and analysis of *ex vivo* intact swine cervix injections of 6% EC-ethanol mixed with iohexol injected with (**a**) extender, (**b**) single needle, and (**c**) multi needle. Images are a top view of the cervix. Scale bars = 5 mm. (**d**) Distribution volume-to-injection ratio analysis. Error bars are standard deviation. No significant differences between groups were observed

The performance of each device was also evaluated in *ex vivo* swine cervical tissue. Experiments used EC-ethanol mixed with a contrast agent, iohexol. The contrast agent appeared white in tissue and was used to visualize the depots. Fig. 4a-c shows representative CT images of EC-ethanol injections in *ex vivo* swine cervical tissue. Fig. 4a and b shows the singular deposits created by the single needle and extender devices, respectively. In contrast, the multi needle creates three separate depots (Fig. 4c) or overlapping depots as shown in Supplementary Fig. 2. When analyzing depot volume, our results indicated that there were no significant differences in distribution volume-to-injection volume ratio. However, the extender’s average ratio (1.1 ± 0.3) was the closest to 1 when comparing all groups, indicating it achieved less leakage away from the injection site. When comparing all groups, the single needle device had the smallest standard deviation (0.74 ± 0.24), and the multi needle injector device had the most variability (0.72 ± 0.36–1.44 ± 0.66 based on the needle).

**Fig. 5:**
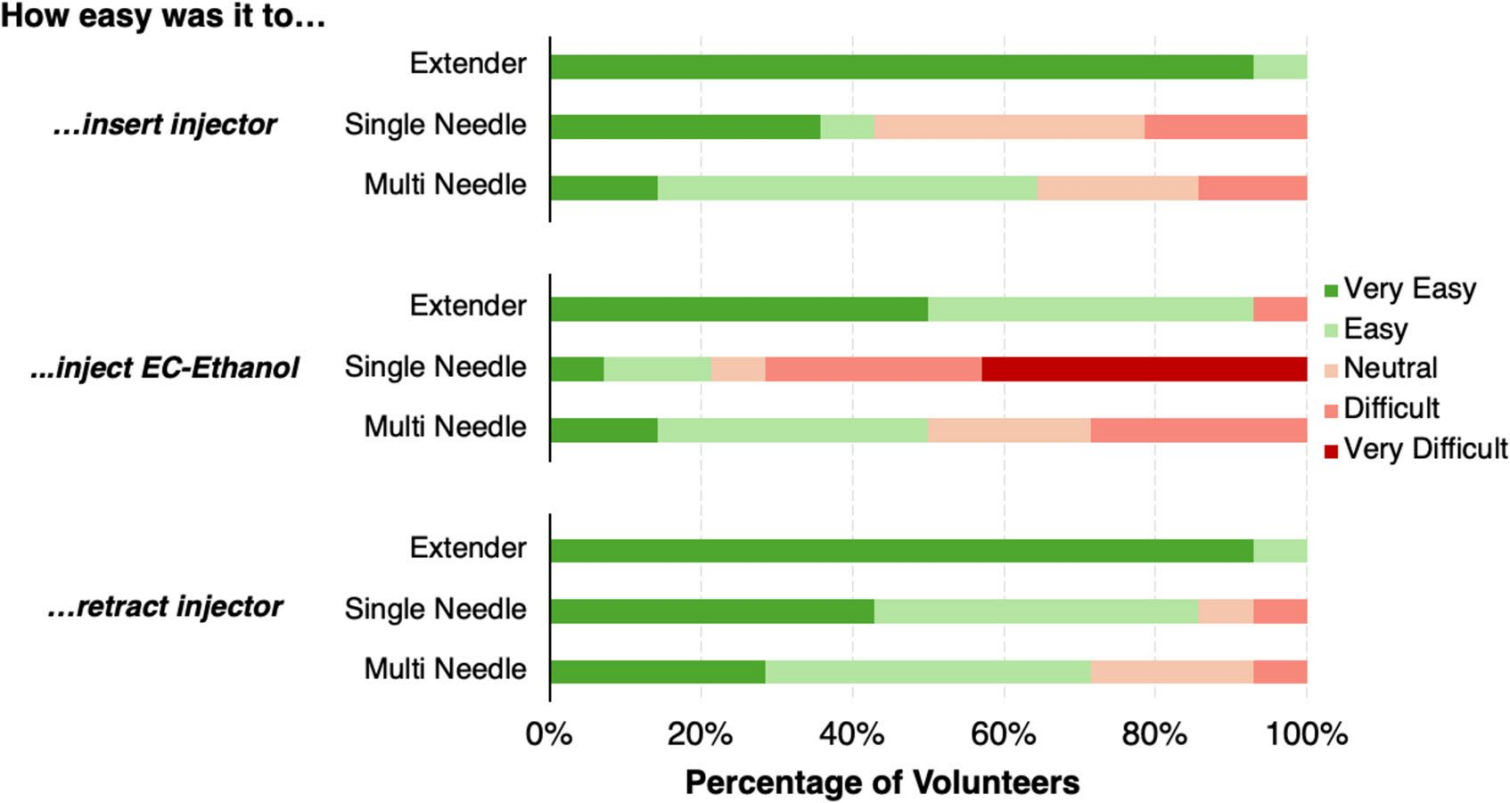
Post-injection questionnaire results from clinicians comparing the usability of the extender, single needle injector, and multi needle injector devices. Questions about the ease of device insertion, 6% EC-Ethanol injection, and device retraction were asked of each participant for each device. *n* = 13 clinicians

After injections, volunteers were asked to evaluate the usability of the device (Fig. 5)**.** The evaluation was based on how easy it was to insert the injector into the cervix, inject EC-ethanol into the cervix, and retract the injector from the cervix. The post-injection questionnaire showed that most clinicians found the extender the easiest to insert, inject EC-ethanol, and retract. More than half of clinicians stated the single needle was difficult or very difficult to insert. The single needle was also rated the most difficult to inject EC-ethanol into the silicone cervix. Over 40% of volunteers stated that it was very difficult to inject EC-ethanol with the single needle device. Approximately, 7% of participants thought that both the single needle and multi needle were difficult to retract from the pelvic model.

**Fig. 6:**
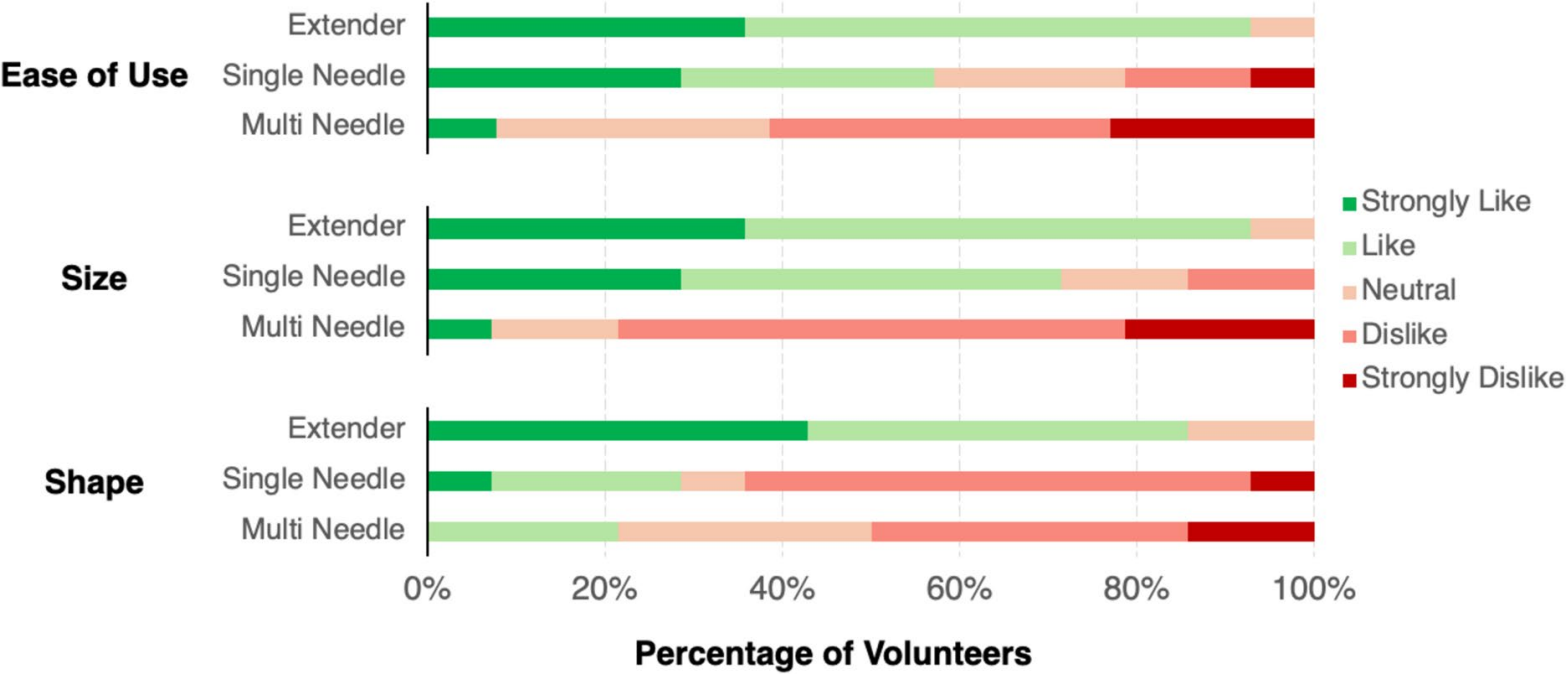
Post-procedure questionnaire results where participants identified their preference in features for each device. Features included ease of use, size, and shape. The participants indicated whether they strongly like, like, neutral, dislike, or strongly dislike the features of each device. *n* = 13 clinicians

In the post-injection questionnaire, participants were also asked about design features of the device including ease of use, size, and shape (Fig. 6), as well as grip and number of needles (Supplementary Fig. 3). For each, clinicians were told to indicate whether they strongly like, like, feel neutral, dislike, or strongly dislike the features. For ease of use, over 80% of volunteers said they strongly liked or liked the extender. Over 60% stated that they strongly disliked or disliked the ease of use of the multi needle injector. Almost 80% of volunteers strongly disliked or disliked the size of the multi needle. In contrast, over 60% strongly liked or liked the size of the extender and single needle devices. We also found that over 80% of clinicians strongly liked or liked the shape of the extender. Most physicians strongly disliked or disliked the shape of the single and multi needle injector devices. Over 80% of physicians strongly liked or liked the grip and the number of needles in the extender design (Supplementary Fig. 3). In contrast, over 20% of participants strongly disliked or disliked the grip and number of needles in the multi needle device design (Supplementary Fig. 3). In written feedback, clinicians cited no complications with the extender device, and one clinician stated that they disliked the “wobbly needle stopper” of the single needle device (Supplementary Table 1). For the multi needle injector, participants stated several problems including decreased visibility of the cervix, concerns about patient intimidation, non-intuitive setup, and others (Supplementary Table 1).

**Fig. 7:**
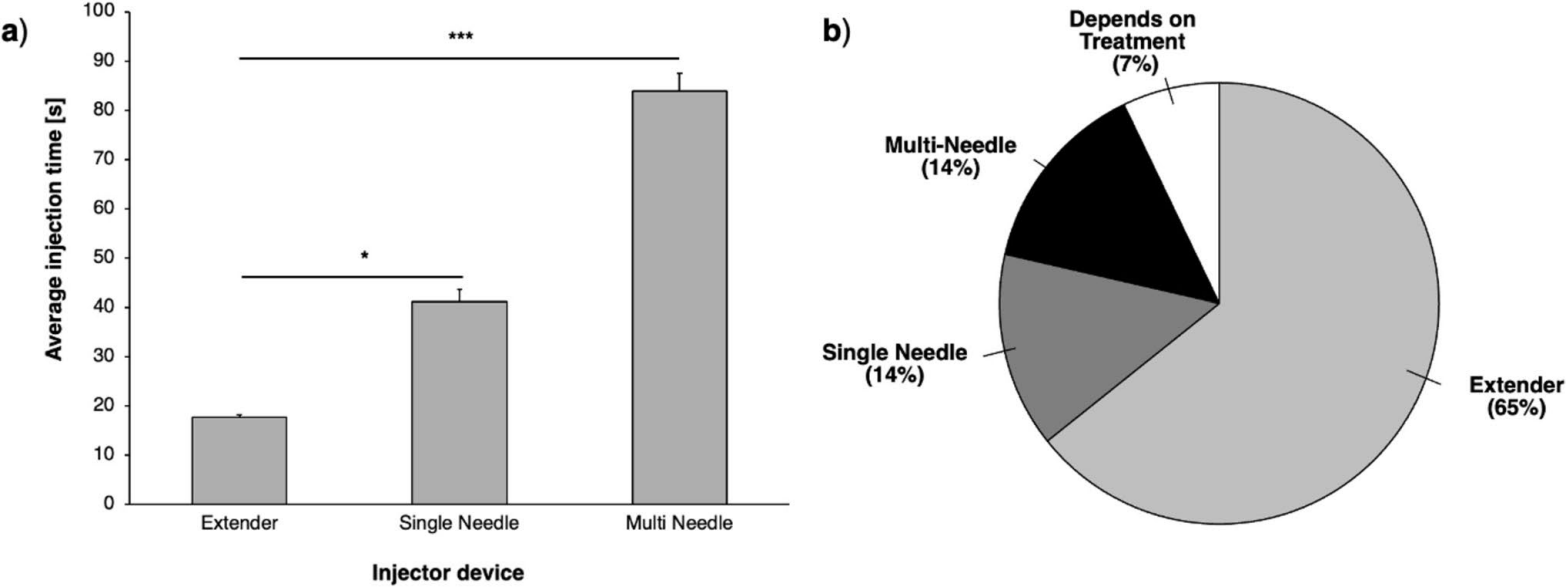
(**a**) The average time to inject 6% EC-ethanol was measured in seconds (sec) for each device used by the participants. Error bars are standard error (*p < 0.05, **p < 0.005, ***p < 0.0005). (**b**) GYN physicians’ overall injector device preference. *n* = 12–13 clinicians

The injection for each clinician was timed. The injection time was defined as when the clinician began to move toward the cervix to inject to the point when the device was fully retracted. Results indicated that the extender needle injector took significantly less time to insert, inject EC-ethanol, and retract the device when compared to both the single needle and multi needle devices. Injections done with the multi needle took the longest time to perform the procedure. Specifically, the extender device took 18 seconds compared to the single needle device with 41 seconds (*p* = 0.03) and the multi needle device with 84 seconds (*p* < 0.0005) (Fig. 7a). When asked about device preference, GYN providers indicated that they preferred the extender needle injector device (65%) over the single needle (14%) and the multi needle (14%) (Fig. 7b). 7% of the participants indicated that their preference would depend on the type of treatment.

## Discussion

Our prior studies found that injection parameters impact the distribution volume of EC-ethanol and are critical to control in order to maximize lesion coverage and minimize off target effects [12, 13]. Leakage can occur through backflow up the needle pathway or crack formation leading to leakage into nearby tissue. Specifically, if the injection depth was too shallow (<13 mm) backflow occurred, and if injection volume exceeded 3 mL, crack formation occurred and EC-ethanol leaked into adjacent tissue [13]. Conversely, injection rate did not need to be controlled if EC concentration was greater than or equal to 6% EC-ethanol. Thus, to facilitate clinical translation and follow a human-centered design approach, we designed three speculum-compatible injectors which address clinical needs: the single needle injector, the extender injector, and multi needle injector (Table 1 and Fig. 1). Here, the performance and usability of each device were determined through bench experiments, *ex vivo* testing, and clinical usability testing. First, the ejection rate and ejection volume of each device were measured by injecting into microcentrifuge tubes to avoid the influence of tissue heterogeneity. Then, depot variability was evaluated in *ex vivo* swine cervix tissue, selected because it is similar in size and morphology to human cervical tissue. Lastly, user feedback was collected from GYN providers who performed injections into a customized pelvic model using our devices (Fig. 2).

Bench experiments indicated that the single needle and extender devices led to similar ranges of depot volume variability (Fig. 3a). However, the extender device achieved moderately faster injection rates (318 mL/hr ± 23) (Fig. 3b), which is likely due to the difference in needle length between the two devices. Specifically, the extender device contained a 2.54 cm 22G needle, while the single needle device used an 8.9 cm 22G needle. Based on the principles of Poiseuille’s law, the increase in needle length likely slowed the volumetric flow rate [17]. Results indicated that the multi needle device led to the most variability in ejection volume (Fig. 3a). The custom syringe of the device was opaque, which prevented the user from visually confirming whether the syringe had been properly loaded with EC-ethanol with no air bubbles. Uneven distribution of the flow of EC-ethanol between the three needles also likely led to higher variability in needle output (Fig. 3).

Results from the *ex vivo* testing showed there were no significant differences in depot volume-to-injection volume ratios between the three devices (Fig. 4). This indicates that all three devices achieved spherical depots with minimal leakage, likely because injection parameters used in this study were informed by previous studies. Specifically, 6% EC-ethanol and 13 mm injection depths were used for all three devices, which has been shown to decrease crack formation and backflow, respectively [13]. While no significant differences were observed, the extender needle device’s average ratio (1.1 ± 0.33) was the closest to 1 when comparing all groups, indicating it had slightly less leakage away from the injection site. The multi needle device had the largest variability in distribution (± 0.36 to 0.66 depending on the needle), likely due to the slight differences in ejection rate between the needles. Lastly, results also showed that the extender, needle 1, and needle 3 groups all had average ratios greater than one. This is likely due to the 5-minute hold time held after each injection during which EC-ethanol and iohexol began to diffuse out into the cervix. Also, because the devices were still inserted into the tissue during the hold time, excess EC-ethanol that was within the device may have continued to flow out of the needle(s).

When tested by clinicians, 65% of volunteers preferred the extender device over the three injectors **(**Fig. 7b). The extender device was also correlated with faster procedure times and favorable rankings of device features and usability (Fig. 5, 6 and 7a). More specifically, over 80% of clinicians strongly liked or liked the extender’s ease of use, size, shape, grip, and number of needles (Fig. 6 and Supplementary Fig. 3). Interestingly, 7% of clinicians felt their device preference depends on treatment type (Fig. 7b). Specifically, those clinicians noted that they would prefer the extender injector for ablating single quadrant lesions but would prefer the multi needle injector for patients with lesions that take up multiple quadrants, which is a sign of more advanced and reoccurring disease [18, 19]. However, based on the average injection time for the extender needle device (18 seconds), multiple injections could be performed within the time frame that it took to deploy the multi needle injector (84 seconds). Additionally, the extender needle allows the provider to select which quadrants to ablate rather than ablating the entire cervix with the multi needle device. Taken together, the extender device is well suited for the administration of the EC-ethanol gel for treatment of both single-lesion ablations and multi-lesion ablations.

While the speculum-compatible devices described here were designed for EC-ethanol injections, they could also aid in several procedures that require clinicians to directly inject medications into the cervix. Potential procedures include paracervical blocks for pain relief and indocyanine green (ICG) injections for lymph node mapping [20–22]. Additionally, the speculum-compatible devices could be used for other intratumoral ablative strategies, such as intratumoral vaccine delivery, which has been found to enhance antitumor response in HPV-associated diseases like CIN and cervical cancer [23]. Our devices’ ability to directly deliver therapeutics into the cervix and control injection parameters to achieve optimal drug distribution could be helpful for these various applications.

Findings from this study will guide clinical implementation of EC-ethanol. Specifically, the extender injector will be applied in a phase 1 clinical study in patients who are scheduled for hysterectomy for benign conditions as was done for thermocoagulation first-in-human trials [24]. We will first validate reprocessing procedures before testing in humans. During the study, the Pocket Colposcope will be used to identify the target area for injections following acetic acid staining. The Pocket Colposcope is a low-cost, portable colposcope found to identify and stage CIN lesions comparably to video colposcopes, the standard of care [25]. The injection will then be performed using the preferred needle extender device; line of sight is preserved, allowing the provider to visually confirm both needle placement and insertion depth. During the injections, providers would rest the device on the speculum to reduce the physician’s hand uncertainty. This will be further investigated in a later phase 1 clinical study. After each procedure, FDA reprocessing techniques will then be followed between patients [26]. More specifically, the single-use needle, syringe, and needle stopper will be discarded, and the extender will be chemically sterilized by submersion into Enzol and Cidex. Injections will be done 24–72 hours before the hysterectomy. This time frame will allow necrosis to form from the EC-ethanol injections [27]. After the hysterectomy is performed, the cervix will be sectioned and stained with hematoxylin and eosin from which we can determine the extent of necrosis induced by different doses of EC-ethanol.

In conclusion, three speculum-compatible device prototypes were developed to deliver EC-ethanol directly into the cervix. Each device was evaluated in benchtop, *ex vivo*, and clinical usability experiments. The extender device achieved small volume variability and the quickest injection time. Additionally, the extender injector was the most preferred by clinicians due to its design, ease of use, and procedure time. Ultimately, this study represents a crucial step toward the clinical implementation of EC-ethanol to treat women with CIN in LMICs and may have additional application for other intracervical injections.

## Supplementary Information

Below is the link to the electronic supplementary material.

Supplementary file1 (TIFF 2004 KB)

Supplementary file2 (TIFF 1637 KB)

Supplementary file3 (TIFF 2430 KB)

Supplementary file5 (TIFF 2472 KB)

Supplementary file5 (TIFF 308 KB)
