# Supplementary figures and images for: Usability of Speculum-Compatible Injection Devices for Administering Ethyl Cellulose-Ethanol Ablation to Treat Cervical Neoplasia in Low- and Middle-Income Countries
Source: Ann Biomed Eng. 2025 Jul 21;53(10):2658–68. doi: 10.1007/s10439-025-03799-8 (PMC12457505; doi:10.1007/s10439-025-03799-8)

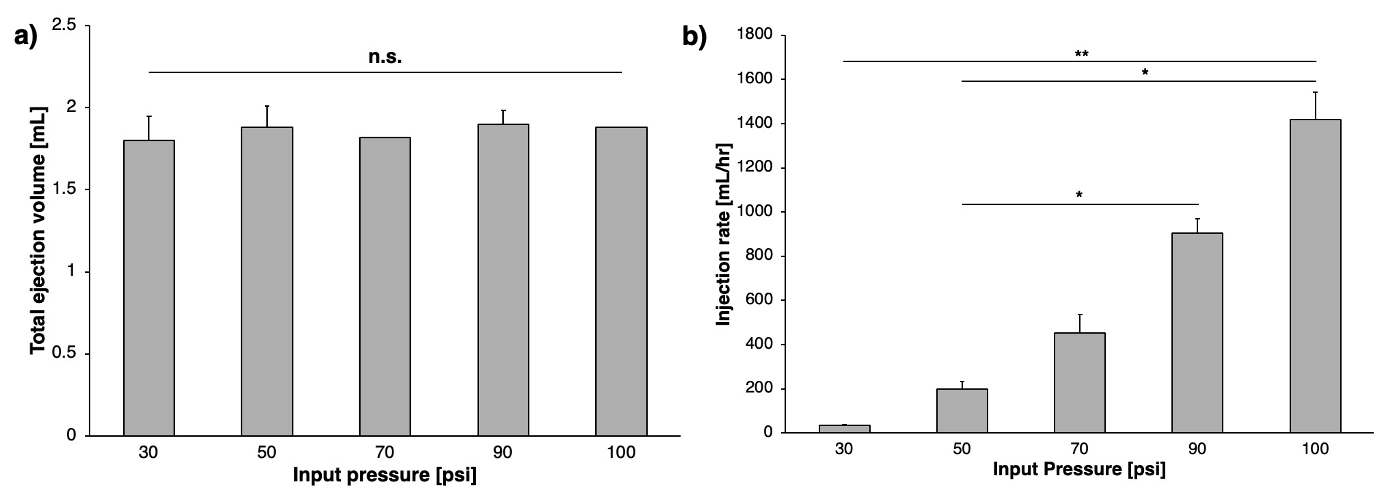

Supplement: Supplementary file 1 — Supplementary file1 (TIFF 2004 KB) [file 10439_2025_3799_MOESM1_ESM.tiff]

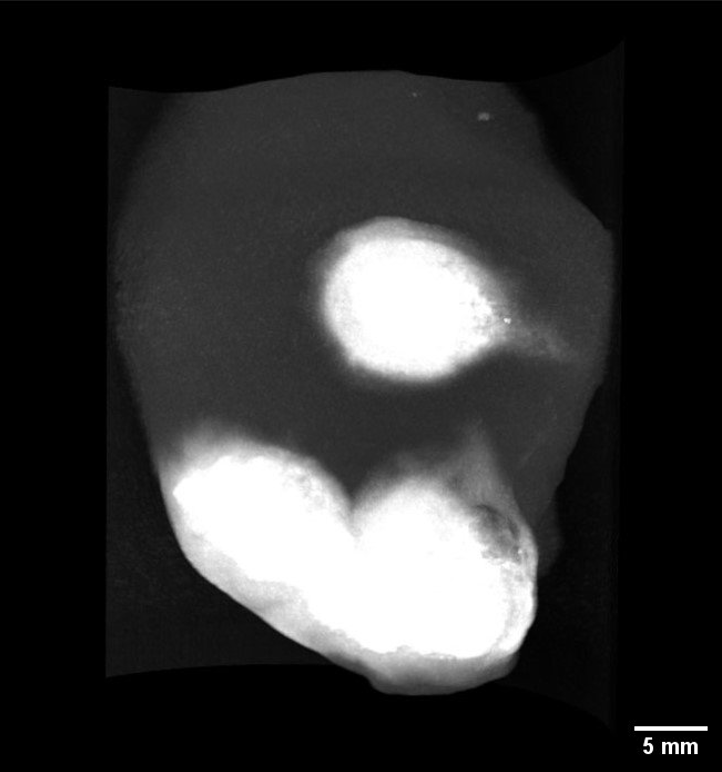

Supplement: Supplementary file 2 — Supplementary file2 (TIFF 1637 KB) [file 10439_2025_3799_MOESM2_ESM.tiff]

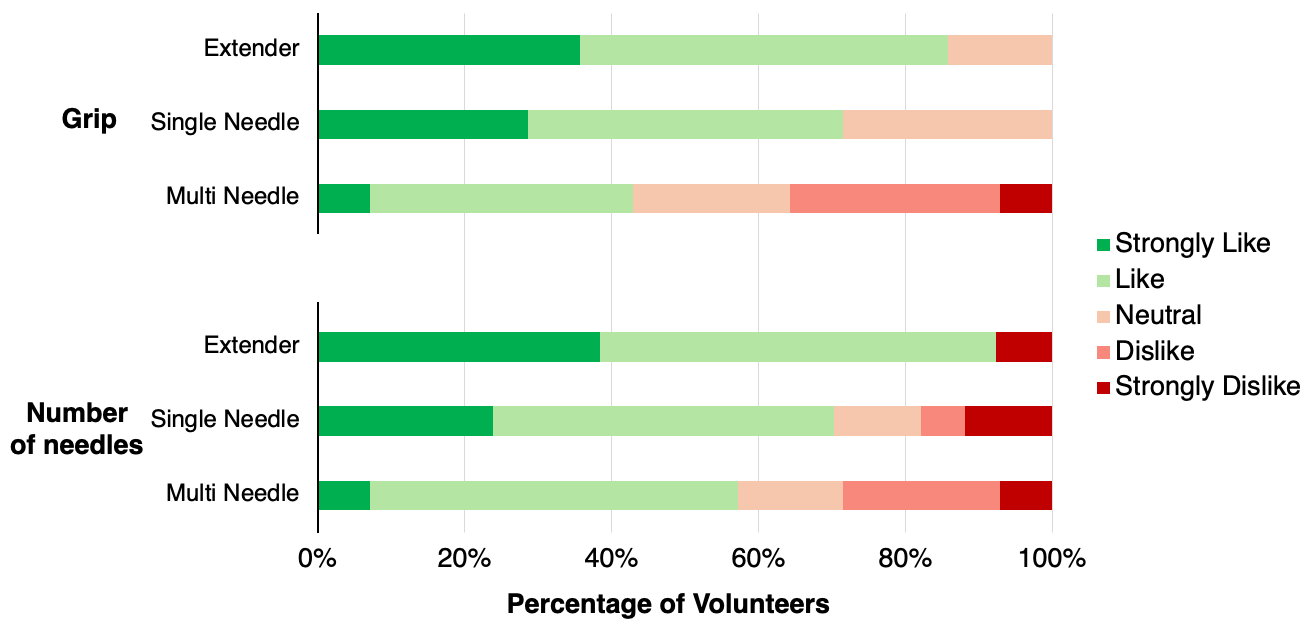

Supplement: Supplementary file 3 — Supplementary file3 (TIFF 2430 KB) [file 10439_2025_3799_MOESM3_ESM.tiff]

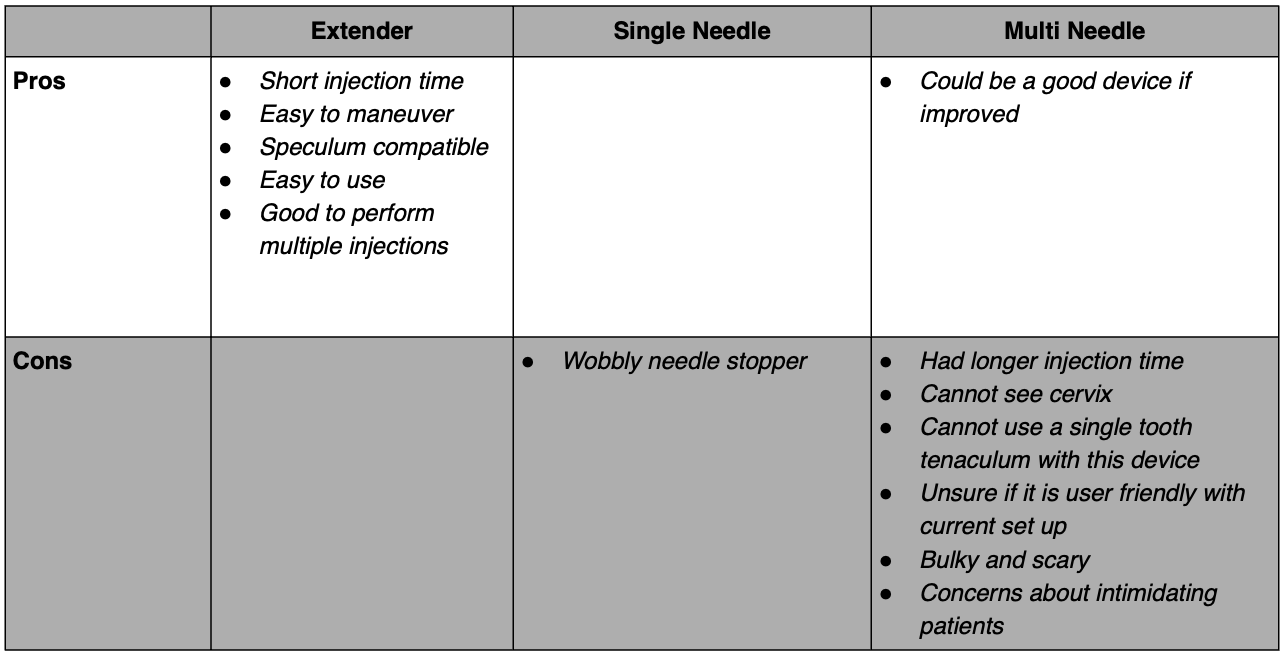

Supplement: Supplementary file 4 — Supplementary file5 (TIFF 2472 KB) [file 10439_2025_3799_MOESM4_ESM.tiff]
