# Supplementary material for: Usability of Speculum-Compatible Injection Devices for Administering Ethyl Cellulose-Ethanol Ablation to Treat Cervical Neoplasia in Low- and Middle-Income Countries
Source: Ann Biomed Eng. 2025 Jul 21;53(10):2658–68. doi: 10.1007/s10439-025-03799-8 (PMC12457505; doi:10.1007/s10439-025-03799-8)

**Usability of Speculum-Compatible Injection Devices for Administering Ethyl Cellulose-Ethanol Ablation to Treat Cervical Neoplasia in Low and Middle-Income Countries**

**Authors:** Taya Lee^1^, Vené Richardson-Powell^1^, Jason Chen^1,2^, David Garvey^1,2^, Venkata Sarojasamhita^1^, Kevin Aroom^2^, Martha O. Wang^2^, Brian Crouch^3,4^, Nimmi Ramanujam^3,4,5,6^, Julie Hurvitz^7^, Jenna Mueller*^1,2,7,8^

^1^ Department of Bioengineering, University of Maryland, College Park, MD, USA.

^2^ Robert E. Fishell Institute for Biomedical Devices, University of Maryland, College Park, MD, USA.

^3^ Department of Biomedical Engineering, Duke University, Durham, North Carolina, USA.

^4^ Calla Health Foundation, Durham, North Carolina, USA.

^5^ Duke Global Health Institute, Duke University, Durham, North Carolina, USA.

^6^ Department of Pharmacology and Cancer Biology, Duke University, Durham, North Carolina, USA.

^7^ Department of Obstetrics, Gynecology & Reproductive Science, University of Maryland School of Medicine, Baltimore, MD, USA.

^8^ Marlene and Stewart Greenebaum Cancer Center, University of Maryland School of Medicine, Baltimore, MD, USA.

**Corresponding Author Information:** Dr. Jenna L. Mueller

**Email Address:** mueller7@umd.edu

**Telephone**: 301-405-8268

**Postal Address:**

3102 A. James Clark Hall

8278 Paint Branch Drive

College Park, MD, US, 20742


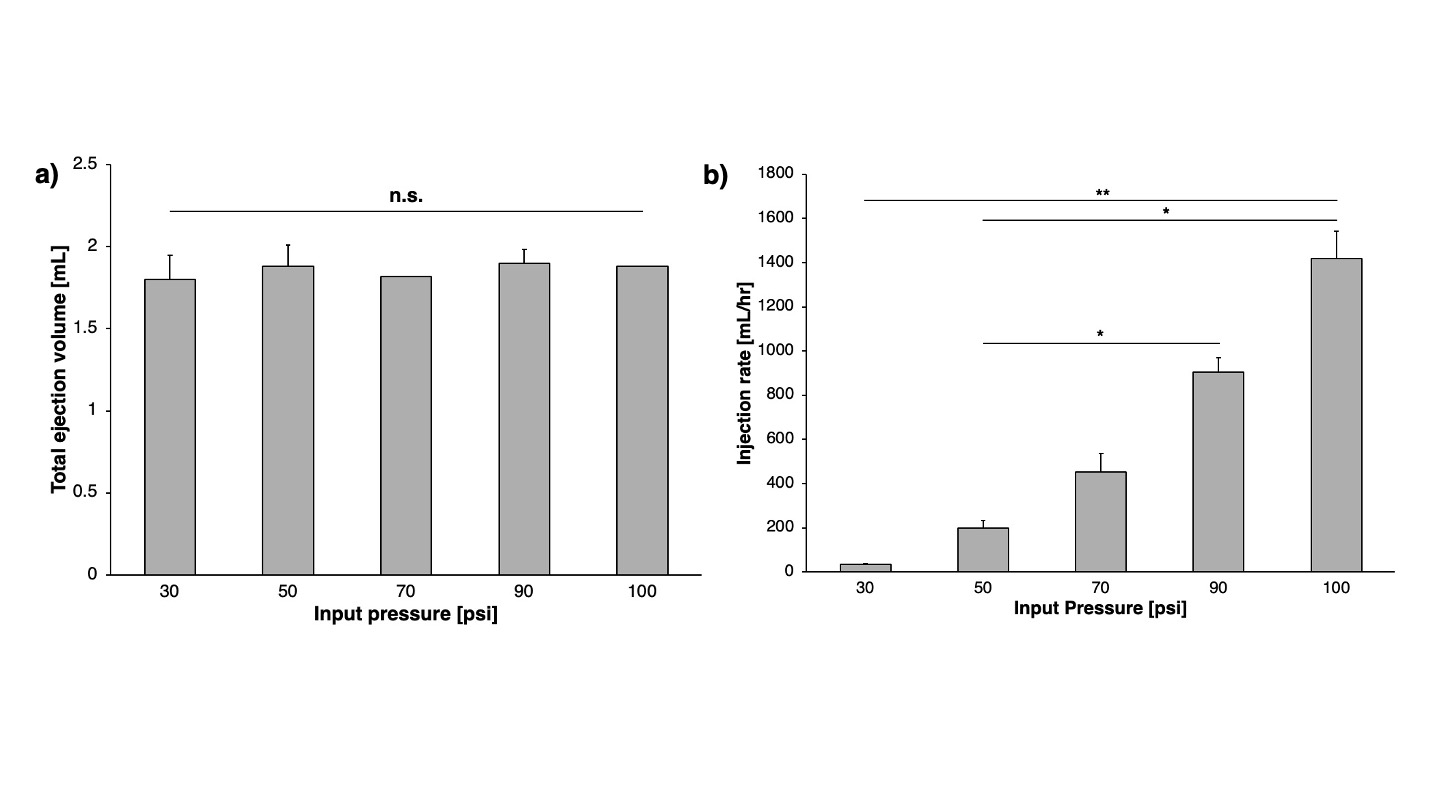


**Supplementary Fig. 1** Multi needle device benchtop experiments at various pressures. **(a)** Ejection volume and **(b)** ejection rate graph for 6% EC-ethanol at different input pressures. Error bars are standard error (*p < 0.05 and **p < 0.005).


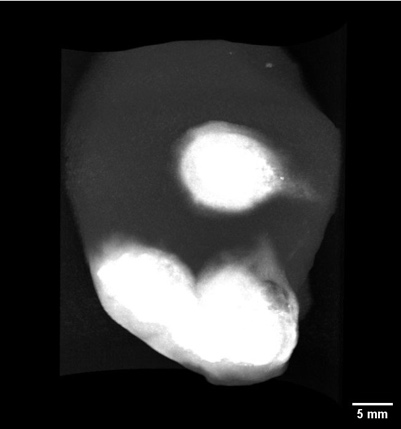


**Supplementary Fig. 2** Mult needle device’s injection volume variability. A representative CT image of overlapping depots in *ex vivo* swine tissue. Scale bar = 5mm.


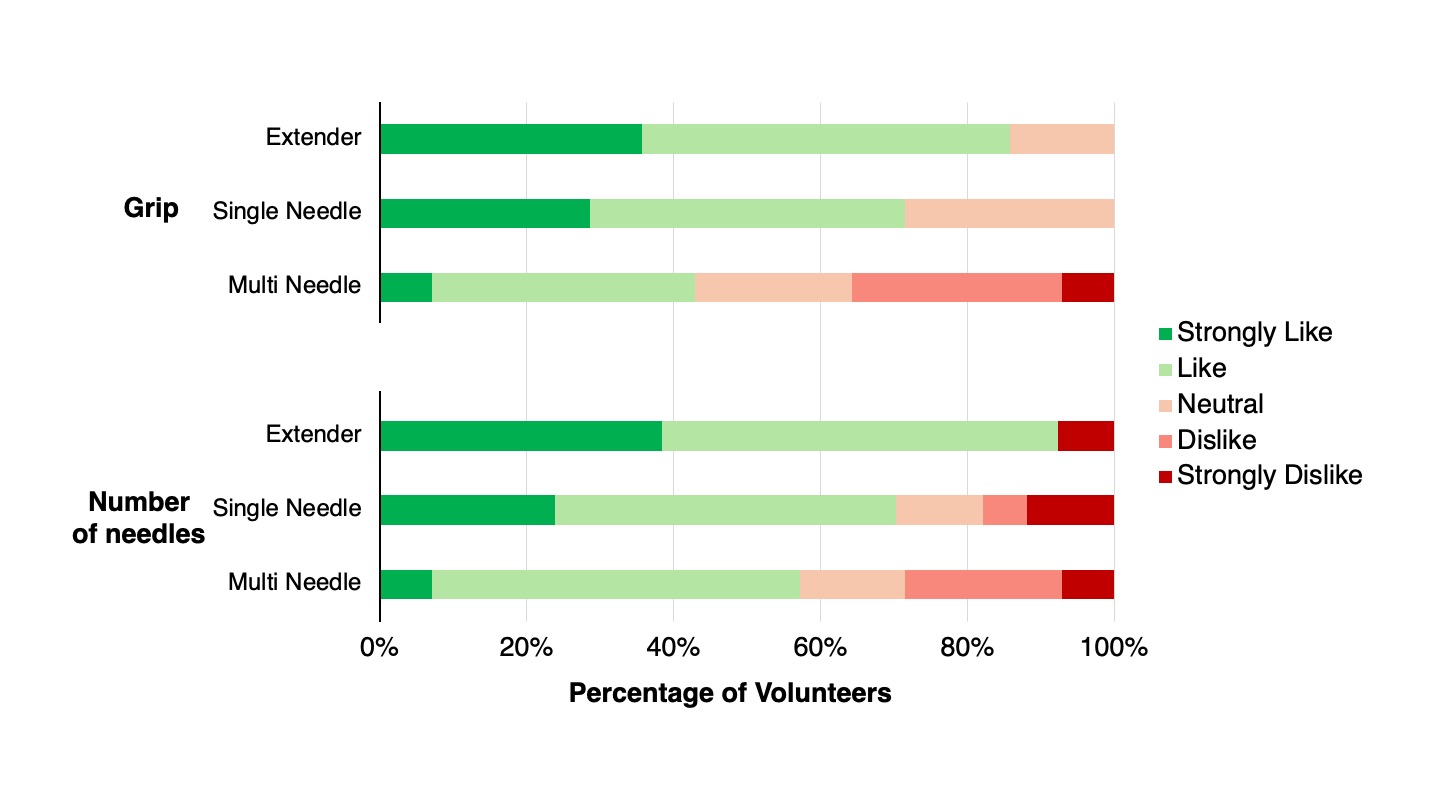


**Supplementary Fig. 3** Additional post procedure questionnaire results where participants identified their preference in grip and number of needles for each device. The participants indicated whether they strongly like, like, neutral, dislike or strongly dislike the features of each device.

**Supplementary Table 1.** Compilation of pros and cons listed by clinicians in response to all three devices


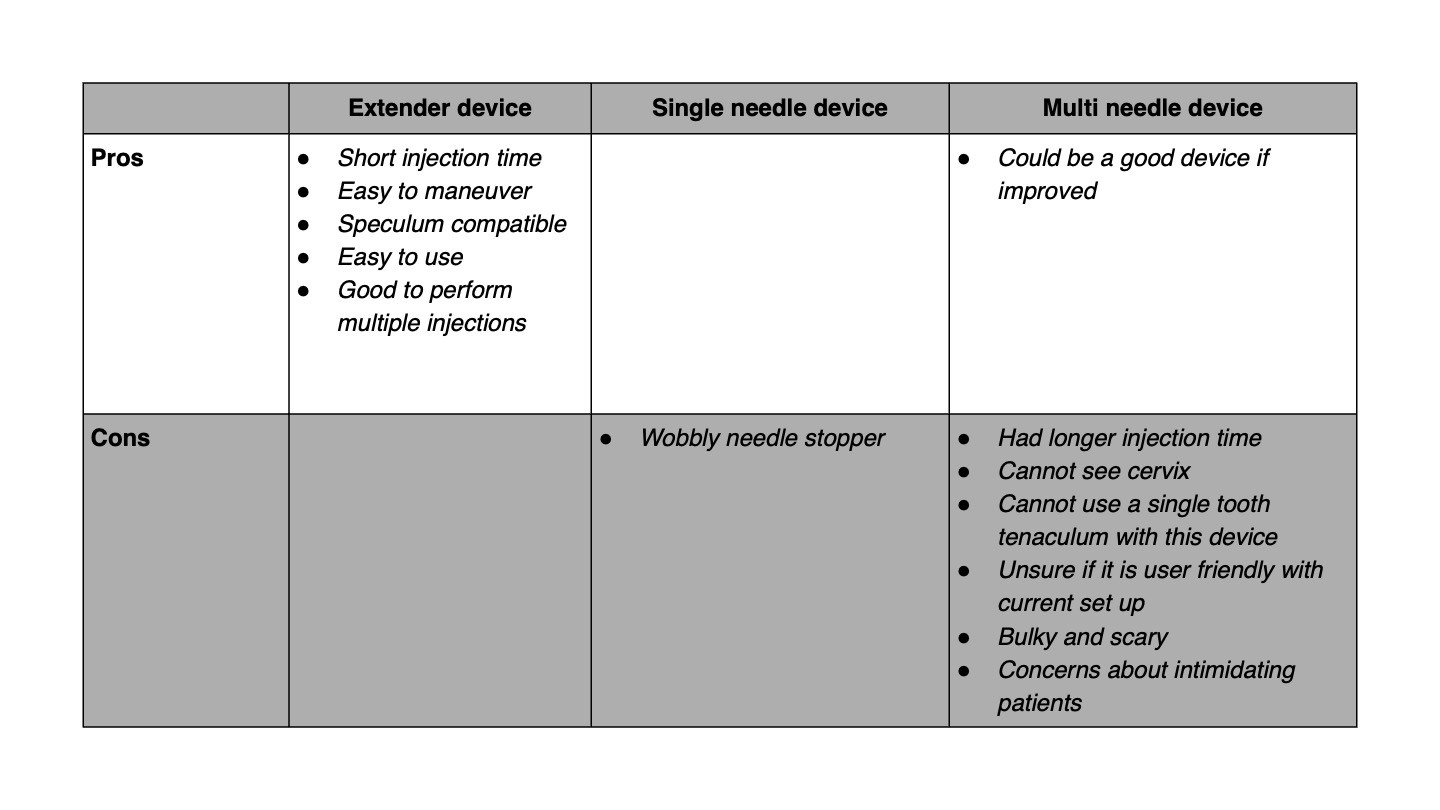

Supplement: Supplementary file 5 — Supplementary file5 (TIFF 308 KB) [file 10439_2025_3799_MOESM5_ESM.docx]
